# Understanding survival comparisons in nonrandomized treatment comparisons for patients with early-stage HCC

**DOI:** 10.1097/HC9.0000000000000281

**Published:** 2023-12-15

**Authors:** Divya Khosla, Gaganpreet Singh, Rakesh Kapoor

**Affiliations:** *Department of Radiotherapy and Oncology, PGIMER, Chandigarh, India*

To the editor,

We would like to congratulate Moon et al.^1^ for their recent publication on thermal ablation versus stereotactic body radiation therapy (SBRT) in HCC. SBRT has evolved over the years and its progress is dependent on technology advancements. Motion management and image-guided delivery with tumor tracking techniques have enabled the delivery of high doses to the tumor with the sparing of surrounding organs. These techniques can decrease the toxicities and improve the therapeutic ratio. There are several issues in the study that need further consideration.

The study was conducted from 2012 to 2018 at 4 different centers. The authors have not elaborated on the quality assurance programs of SBRT on which the results depend. Practice may vary among the centers, depending on the available infrastructure. The dose and fractionation might also be different between different centers. There is wide variation in the biologically effective dose ranging from 20 to 180 Gy with a mean of 88 Gy. There was an increase in the Child-Pugh score observed with SBRT after 3 months but was not significant at 6 months. This may have been due to a larger volume of irradiation in patients undergoing SBRT because of relatively larger treated lesions. It should be noted that in the recently published meta-analysis by Rim et al,^2^ ablative radiotherapy not only resulted in similar oncological outcomes compared to radiofrequency ablation but also was more effective for larger lesions and specific locations.

In a propensity-matched multinational study of 2064 patients of HCC from 5 countries, there was no difference in overall survival between the radiofrequency ablation and SBRT arms.^3^ Though Moon et al adjusted for various important confounders like treatment center, tumor size and number, AFP levels, Eastern Cooperative Oncology Group (ECOG) performance status, and the number of prior off-target treatments, some pertinent questions still remain. The SBRT arm had a history of more prior treatments which could also highlight relatively biologically aggressive disease in the SBRT group. In addition, and as the authors highlight, patients treated by SBRT had more comorbidities than those treated by ablation, reflecting the current status and the use of SBRT in real-world settings. Most of the studies are potentially impacted by selection bias and despite adjustment, we suspect this heterogeneity could not be overcome resulting in survival differences between SBRT and other ablative treatment methods.

Overall survival comparisons should be interpreted with caution. These outcomes can be influenced by noncancer deaths, subsequent therapies given after the failure of primary therapy, and whether the subsequent therapies work equally in the two treatment arms. While considering overall survival as the endpoint, it is important to note the other treatments patients received as the current management of HCC is multimodal. Together these factors might have contributed to the divergence between local control and overall survival.
